# Supplementary figures and images for: Intracellular niche-specific profiling reveals transcriptional adaptations required for the cytosolic lifestyle of Salmonella enterica
Source: PLoS Pathog. 2021 Aug 30;17(8):e1009280. doi: 10.1371/journal.ppat.1009280 (PMC8432900; doi:10.1371/journal.ppat.1009280)

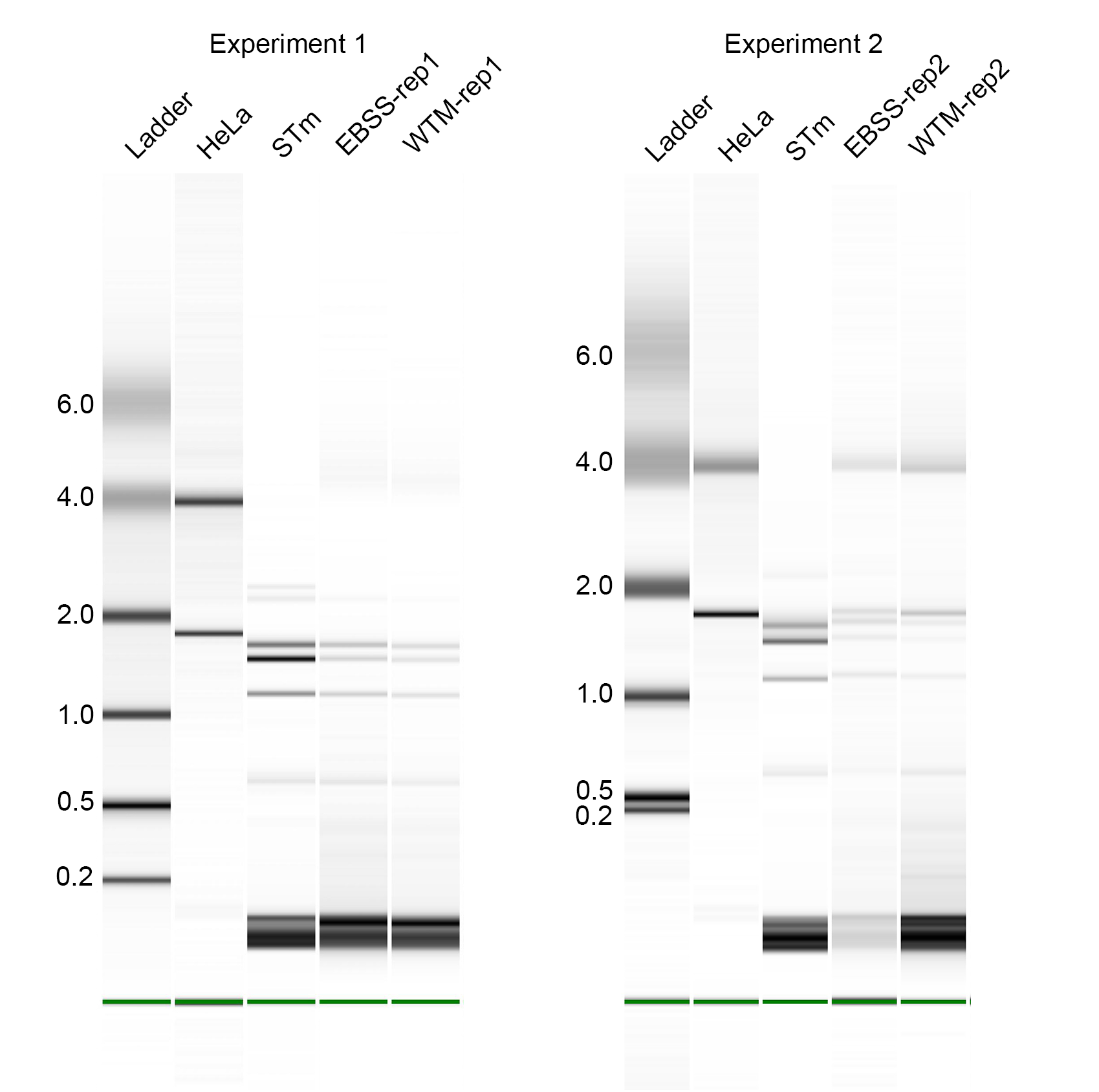

Supplement: S1 Fig — Total RNA was extracted from HeLa epithelial cells (HeLa), wild-type S. Typhimurium SL1344 grown to late log-phase in LB-Miller broth (STm), S. Typhimurium isolated from Earle’s balanced salt solution (EBSS)-treated cells at 8 h p.i. or S. Typhimurium isolated from wortmannin (WTM)-treated cells at 8 h p.i. RNA quality was analyzed by electrophoretic separation using an Agilent Bioanalyzer 2100. Ladder sizes shown in kb. (TIF) [file ppat.1009280.s001.tif]

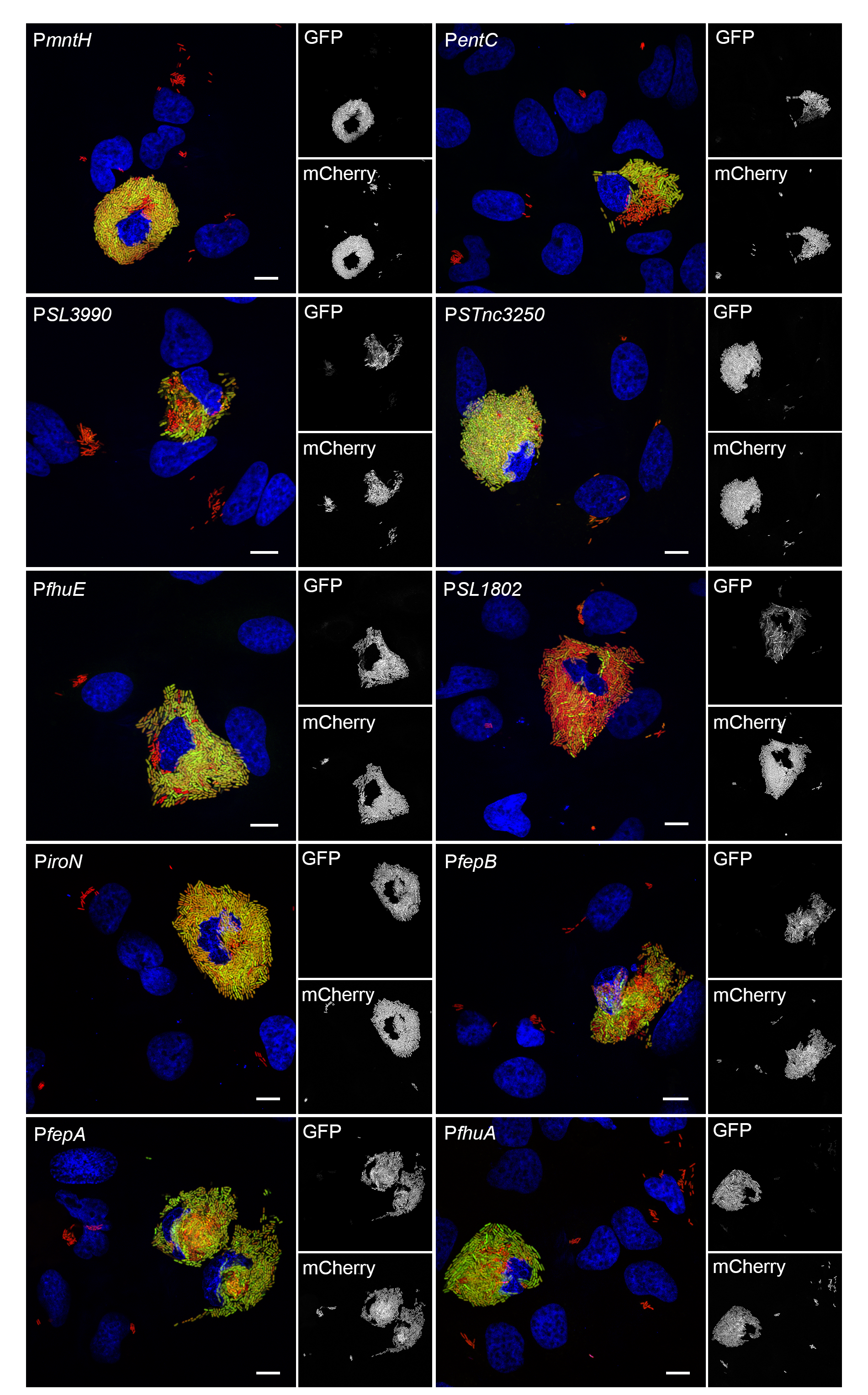

Supplement: S3 Fig — Epithelial cells were infected with mCherry-S. Typhimurium harboring gfpmut3 transcriptional reporters. At 8 h p.i., cells were fixed & stained with Hoechst 33342 to detect DNA. Representative confocal microscopy images show induction of mntH, entC, SL3990, STnc3250, fhuE, SL1802, iroN, fepB, fepA and fhuA promoters in cytosolic bacteria. Green = transcriptional reporter, red = S. Typhimurium, blue = DNA. Scale bars = 10 μm. (TIF) [file ppat.1009280.s003.tif]

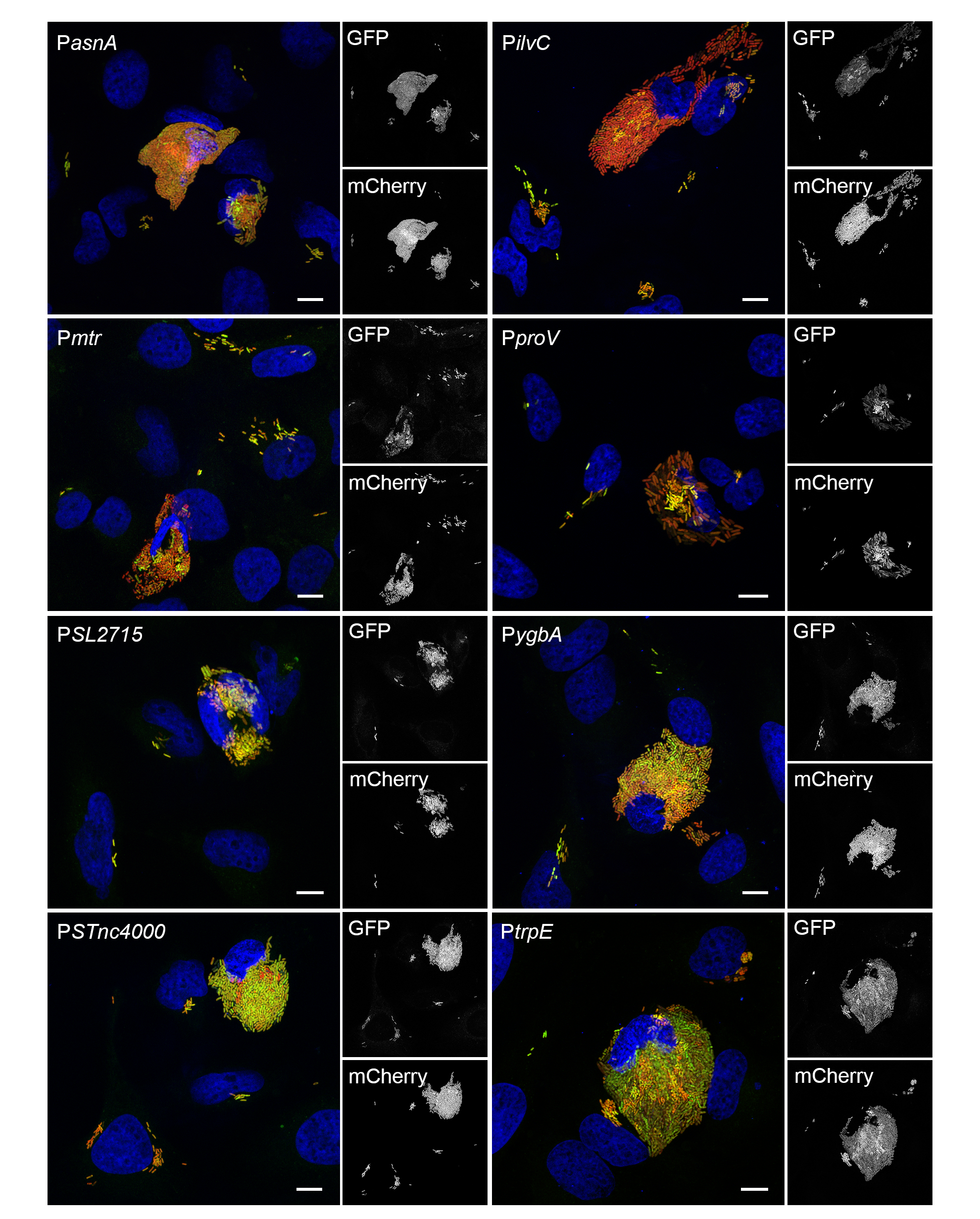

Supplement: S4 Fig — Epithelial cells seeded on coverslips were infected with mCherry-S. Typhimurium harboring gfpmut3 transcriptional reporters. At 8 h p.i., cells were fixed and stained with Hoechst 33342 to detect DNA. Representative confocal microscopy images show equivalent expression of asnA, ilvC, mtr, proV, SL1344_2715, ygbA, STnc4000 and trpE promoters in vacuolar and cytosolic bacteria. Green = transcriptional reporter, red = S. Typhimurium, blue = DNA. Scale bars are 10 μm. (TIF) [file ppat.1009280.s004.tif]

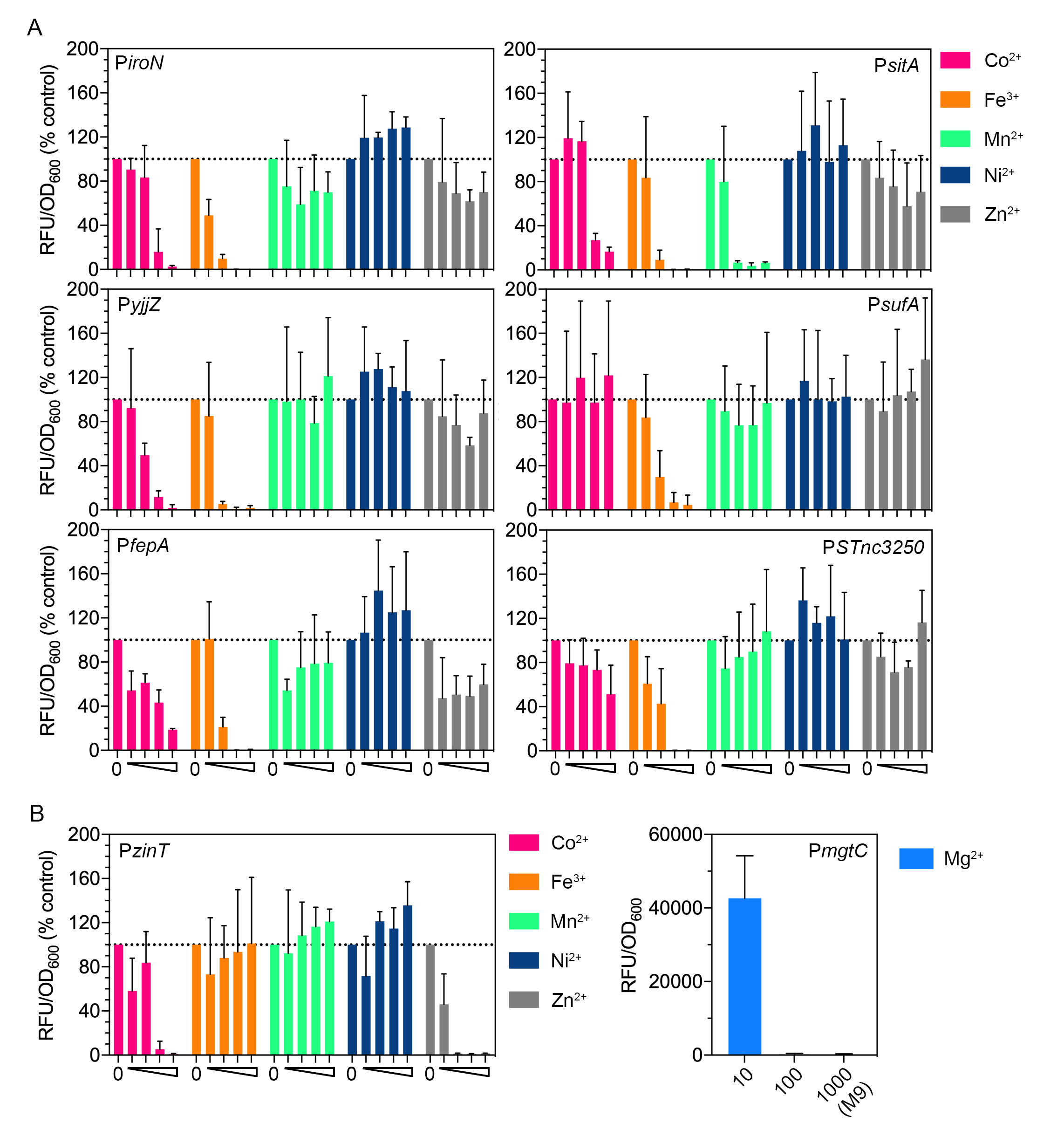

Supplement: S5 Fig — (A). GFP fluorescence in mCherry-S. Typhimurium harboring PiroN-gfpmut3, PsitA-gfpmut3, PyjjZ-gfpmut3, PsufA-gfpmut3, PfepA-gfpmut3 or PSTnc3250-gfpmut3 reporter plasmids. Bacteria were grown shaking overnight at 37°C for 16 h in M9-supplemented media containing increasing concentrations of CoCl2, FeCl3, MnCl2, NiCl2 or ZnCl2 (0.1 μM, 1 μM, 10 μM or 100 μM). No added cation (0) served as the control. The relative fluorescence units (RFU) were normalized to OD600 and expressed as a percentage of control (set to 100%). Background GFP fluorescence of mCherry-S. Typhimurium (no reporter) was subtracted from all values. n≥3 independent experiments. (B) GFP fluorescence in mCherry-S. Typhimurium harboring PzinT-gfpmut3 or PmgtC-gfpmut3 plasmids. Growth and analysis of zinT expression was as described in (A). Standard M9 minimal media contains 1 mM MgSO4 which completely represses mgtC expression. The effect of Mg2+ concentration on mgtC expression was therefore assessed in M9-supplemented media containing decreasing amounts of MgSO4 i.e. 1000 μM (standard), 100 μM and 10 μM. Mg2+ concentrations lower than 10 μM impacted bacterial growth. n = 3 independent experiments. (TIF) [file ppat.1009280.s005.tif]

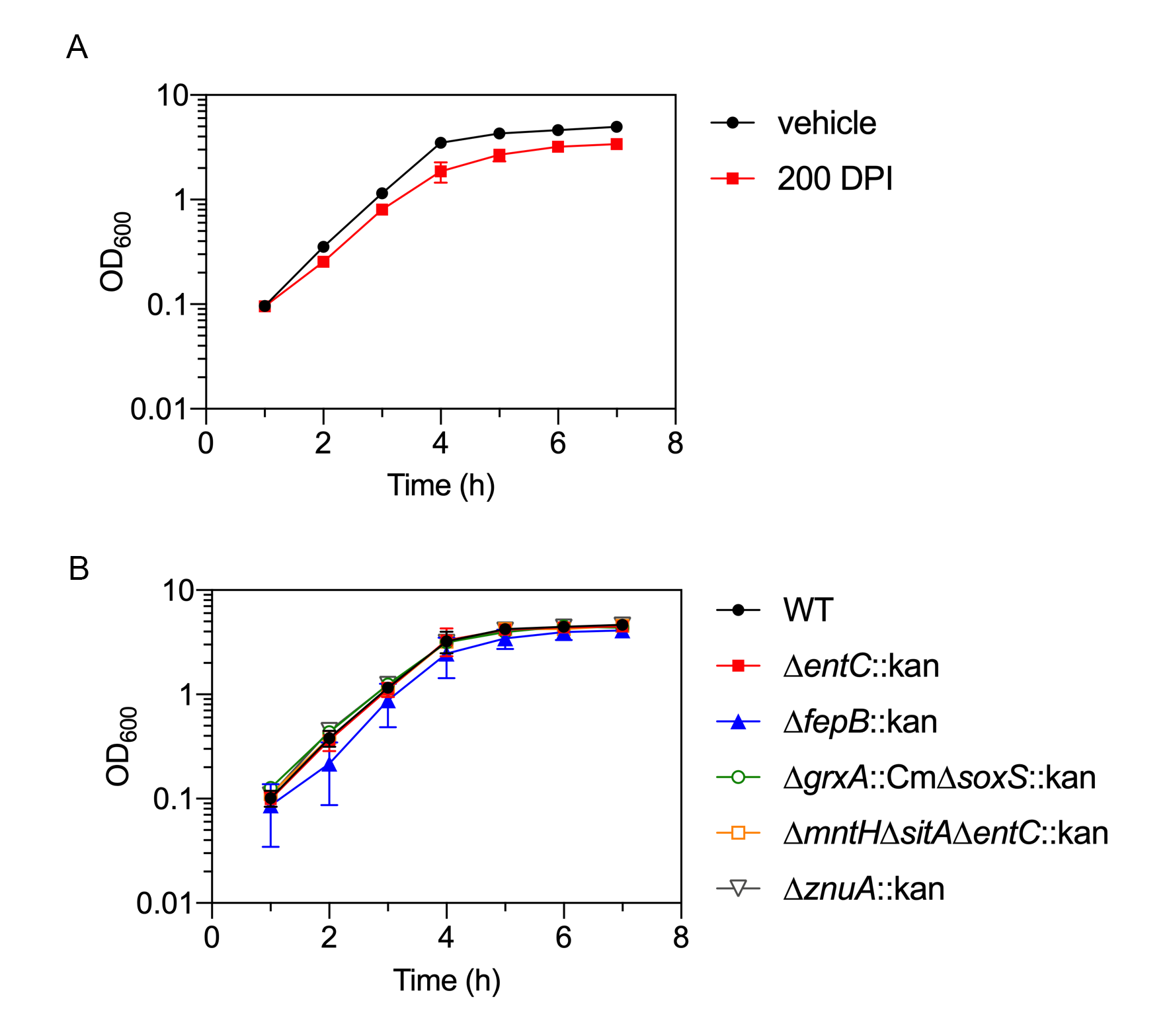

Supplement: S6 Fig — (A) Overnight cultures of wild-type S. Typhimurium were subcultured 1:100 in LB-Miller broth in the presence of 200 μM 2,2’-dipyridyl (DPI) or vehicle (ethanol) control. Growth was measured every hour by optical density at 600 nm (OD600). n = 3 independent experiments. (B) Overnight cultures of wild-type S. Typhimurium or the indicated deletion mutants were subcultured 1:100 in LB-Miller broth. Growth was measured every hour by optical density at 600 nm (OD600). n = 3 independent experiments. (TIF) [file ppat.1009280.s006.tif]

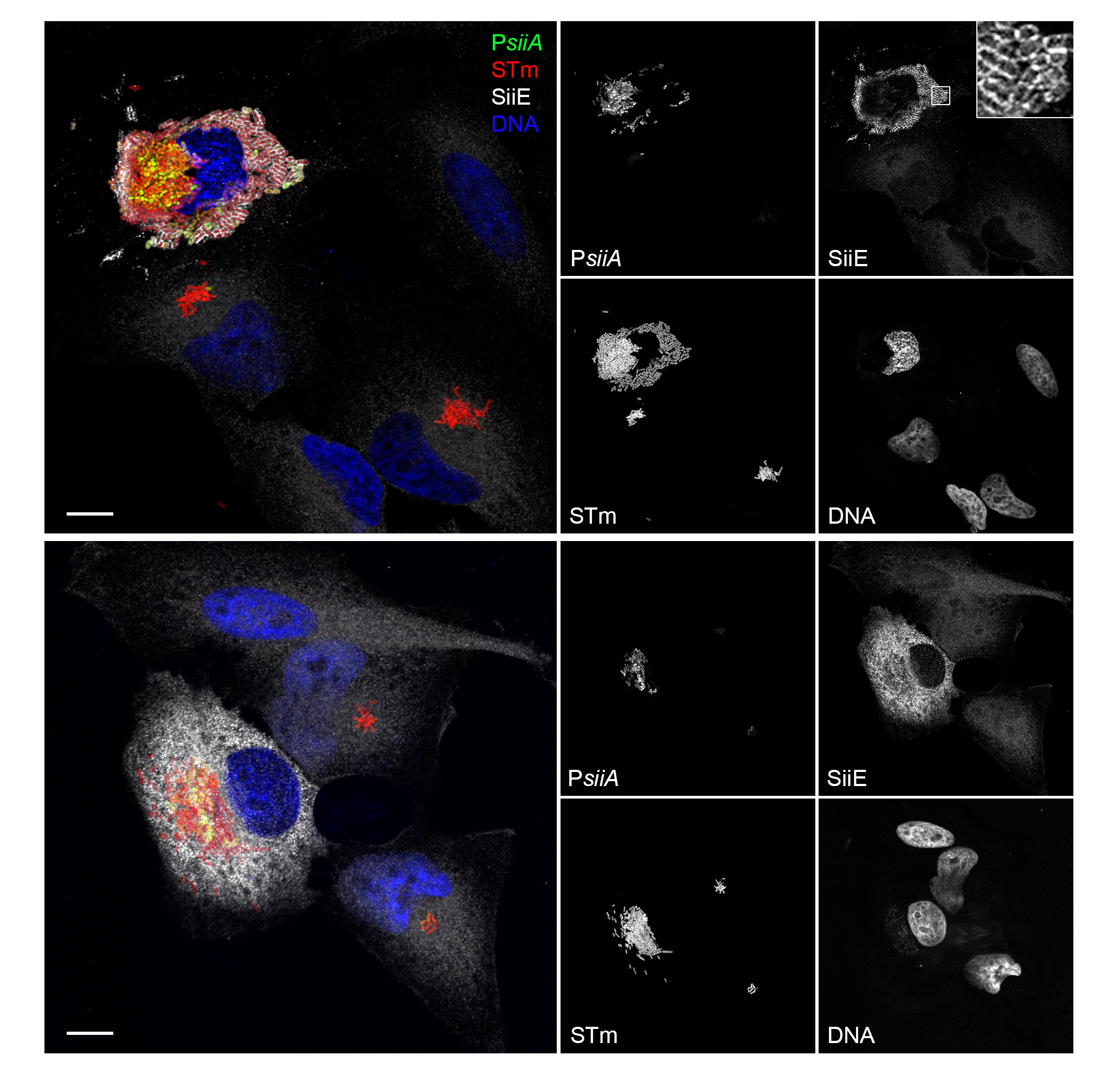

Supplement: S7 Fig — Epithelial cells seeded on coverslips were infected with mCherry-S. Typhimurium harboring a PsiiA-gfpmut3 transcriptional reporter. At 8 h p.i., cells were fixed and immunostained with polyclonal antibodies directed against SiiE. DNA was stained with Hoechst 33342. Representative confocal microscopy images show SiiE attached to (upper panel) or secreted by (lower panel) cytosolic bacteria. Inset shows enlargement of boxed area. Green = PsiiA-gfpmut3 reporter, red = S. Typhimurium (STm), white = SiiE, blue = DNA. Scale bars are 10 μm. (TIF) [file ppat.1009280.s007.tif]

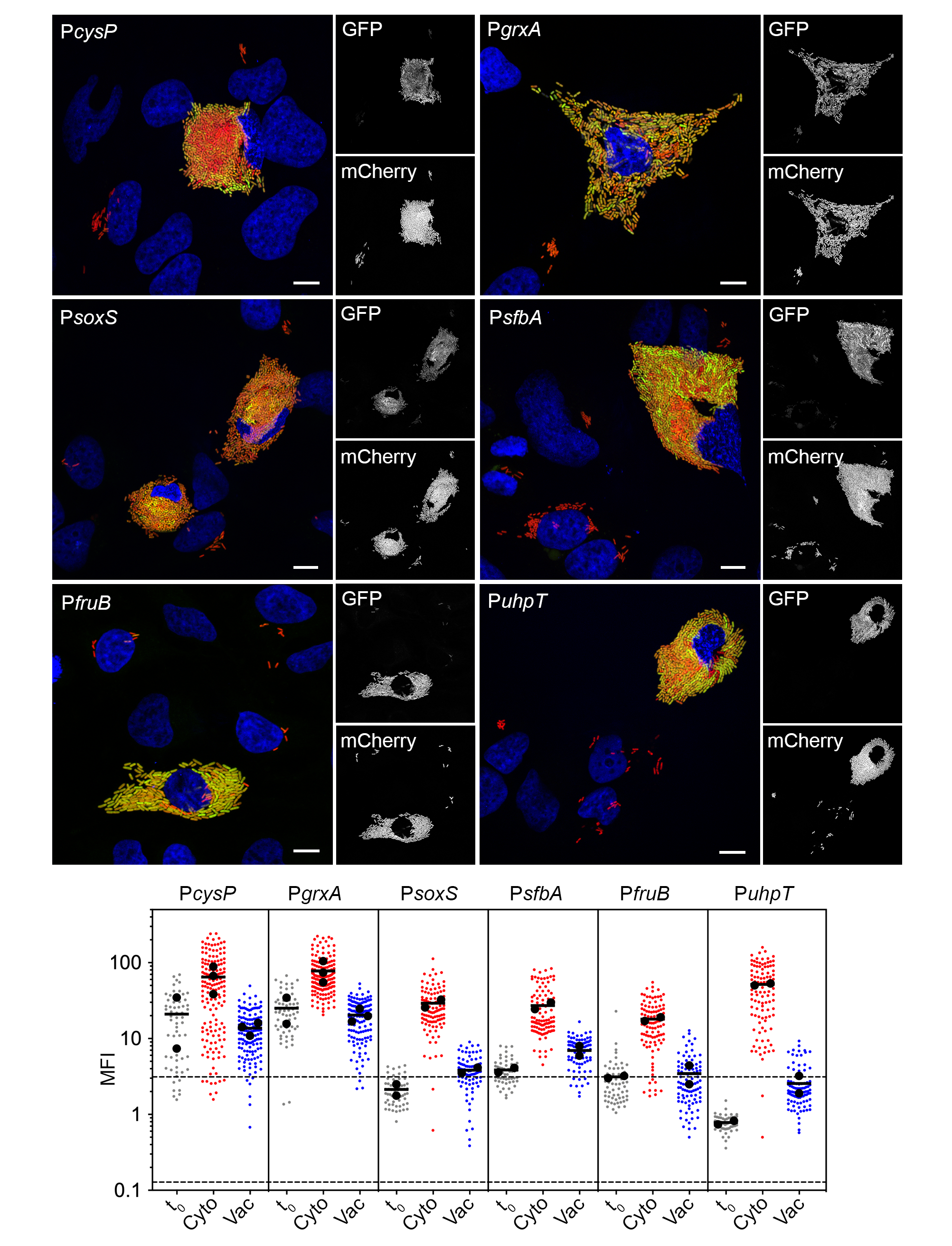

Supplement: S8 Fig — Upper panels: Epithelial cells were infected with mCherry-S. Typhimurium harboring gfpmut3 transcriptional reporters. At 8 h p.i., cells were fixed & stained with Hoechst 33342 to detect DNA. Representative confocal microscopy images show induction of cysP, grxA, soxS, sfbA, fruB and uhpT promoters in cytosolic bacteria. Green = transcriptional reporter, red = S. Typhimurium, blue = DNA. Scale bars are 10 μm. Lower panel: Quantification of the MFI of GFP signal by fluorescence microscopy and ImageJ. Small dots represent individual bacteria; large dots indicate the mean of each experiment; horizontal bars indicate the average of 2–3 experiments. Acquisition parameters (exposure time and gain) were set-up using PcysP-gfpmut3 (the highest GFP signal intensity) and these same parameters were applied throughout. Dashed lines indicate the range of background fluorescence in the GFP channel measured for mCherry-S. Typhimurium (no reporter). (TIF) [file ppat.1009280.s008.tif]
