# Supplementary material for: Intracellular niche-specific profiling reveals transcriptional adaptations required for the cytosolic lifestyle of Salmonella enterica
Source: PLoS Pathog. 2021 Aug 30;17(8):e1009280. doi: 10.1371/journal.ppat.1009280 (PMC8432900; doi:10.1371/journal.ppat.1009280)
Supplement: S2 Fig — Each arrow represents an individual gene to scale within each PAI. The different islands are also scaled against each other. The color of each arrow represents relative gene expression–red arrows depict genes up-regulated in the cytosol (≥1.40-fold change WTM/EBSS), blue are genes up-regulated in the vacuole (≥1.40-fold change EBSS/WTM), yellow are genes with unchanged expression (0.72–1.39-fold change) and grey arrows are genes with a TPM value <10 and considered not expressed. See S1 Dataset for the entire data set. Adapted from Srikumar et al. [43]. (PPTX) [file ppat.1009280.s002.pptx]

## Slide 1
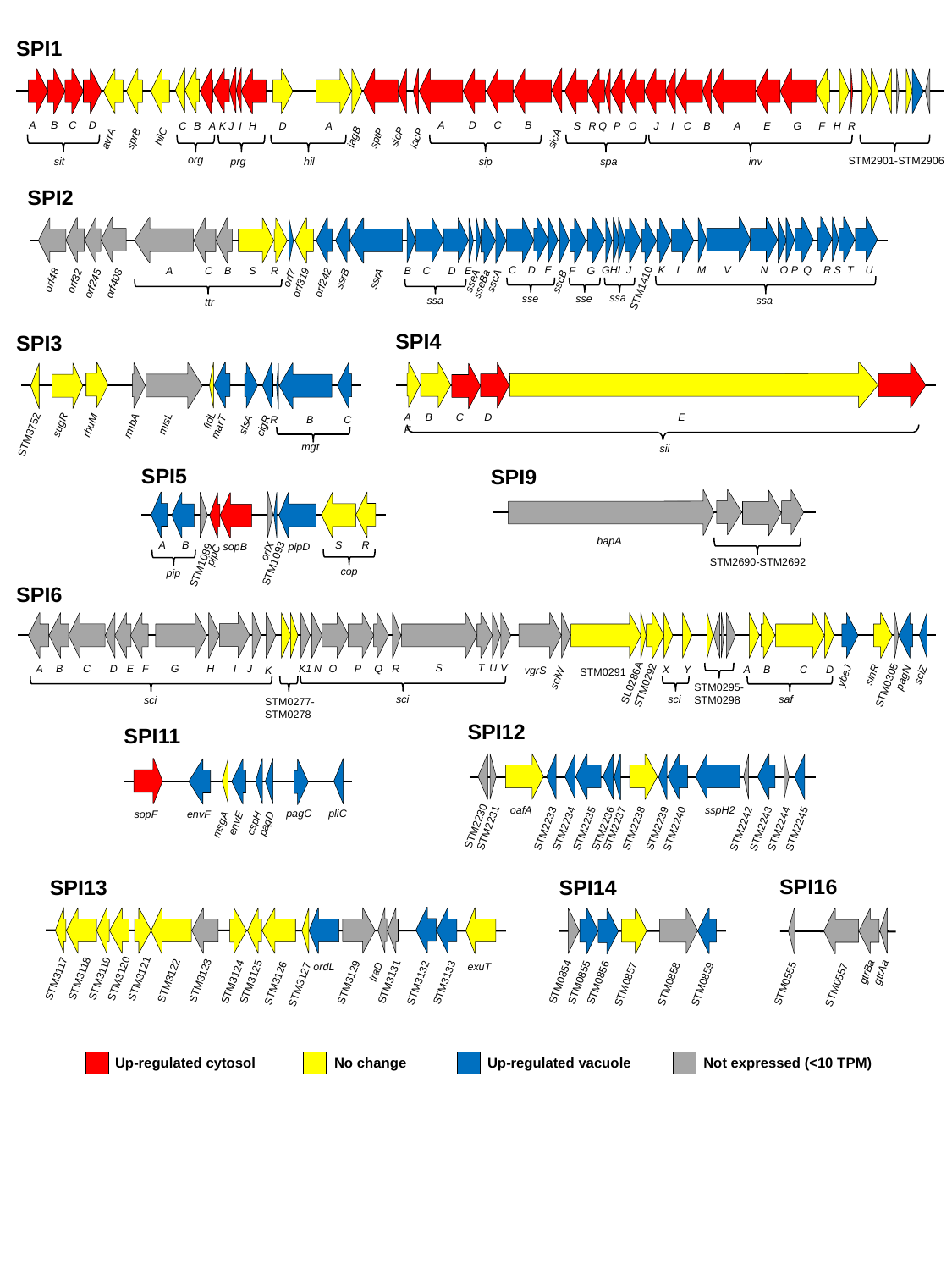

SPI1
D
A
C
B
B
D
C
A
iagB
sicP
iacP
sptP
hilC
avrA
sprB
R
B
K
J
I
H
D
S
R
Q
P
O
J
I
C
B
A
E
G
F
H
C
A
A
sicA
STM2901-STM2906
org
inv
sit
prg
hil
sip
spa
SPI2
K L M V N O P Q R S T U
GHI J
C D E
B C D E
F G
A C B S R
orf7
ssrA
ssrB
orf48
sseA
orf32
sscA
sscB
orf319
orf242
orf245
orf408
sseBa
STM1410
ssa
sse
sse
ssa
ssa
ttr
SPI4
A B C D E F
sii
SPI3
R
B
C
misL
slsA
sugR
cigR
rhuM
rmbA
marT
STM3752
mgt
fidL
SPI5
A
B
pip
R
S
cop
pipD
sopB
orfX
pipC
STM1093
STM1089
SPI9
bapA
STM2690-STM2692
SPI6
S
T
U
V
P
Q
R
K1
N
O
A
B
C
D
E
F
G
H
I
J
K
sci
Y
A
B
C
D
X
STM0291
STM0292
vgrS
sciW
pagN
sciZ
ybeJ
sci
saf
sci
sinR
SL0286A
STM0305
STM0295-
STM0298
STM0277-
STM0278
SPI12
oafA
sspH2
STM2230
STM2231
STM2240
STM2242
STM2243
STM2244
STM2245
STM2233
STM2234
STM2235
STM2236
STM2237
STM2238
STM2239
SPI11
pliC
pagC
envF
envE
cspH
pagD
msgA
sopF
SPI16
gtrBa
gtrAa
STM0555
STM0557
SPI13
STM3120
STM3117
STM3118
STM3119
STM3121
STM3124
STM3125
STM3127
STM3131
STM3133
exuT
ordL
iraD
STM3122
STM3123
STM3129
STM3132
STM3126
SPI14
STM0854
STM0855
STM0856
STM0858
STM0857
STM0859
Up-regulated cytosol
No change
Up-regulated vacuole
Not expressed (<10 TPM)
