# Supplementary material for: Intracellular niche-specific profiling reveals transcriptional adaptations required for the cytosolic lifestyle of Salmonella enterica
Source: PLoS Pathog. 2021 Aug 30;17(8):e1009280. doi: 10.1371/journal.ppat.1009280 (PMC8432900; doi:10.1371/journal.ppat.1009280)
Supplement: S1 Table — (DOCX) [file ppat.1009280.s009.docx]

**S1 Table: Oligonucleotides used to construct gene deletion mutants**

| **Name** | **Sequence (5’ to 3”)** | **Gene deletion** |
| --- | --- | --- |
| Kpn-sitA | GGGGTACCTCGCCTGTTGCGACTGAAAG | ∆*sitA* |
| sitA OL-F | GATACT**ATG**ACGAATGACGGACTGAGGAGTCAACAA**TGA** | ∆*sitA* |
| sitA OL-R | GACTCCTCAGTCCGTCATTCGT**CAT**AGTATCCCTCGCAACAATG | ∆*sitA* |
| Xma-sitA | CCCCCCGGGAAATAAAGGGGCGCTCGTC | ∆*sitA* |
| Xma-mntH3 | CCCCCCGGGCGGCGAGCGGAAGCCGTTTC | ∆*mntH* |
| mntH OL-F | AAGGCTATGTTTTTGTTGTCA**TAA**AGACAGAAAGATAGCAGGCC | ∆*mntH* |
| mntH OL-R | CTGTCT**TTA**TGACAACAAAAACATAGCCTTTGCTATGTTTCATG | ∆*mntH* |
| Xba-mntH2 | GCTCTAGAATGGTCATATACGCGCCCAC | ∆*mntH* |
| SL4483-yjjZ-Xba | GCTCTAGATAACATGTTGAATAGCGATTC | ∆*yjjZ* |
| SL4483-yjjZ OL-F | GAAACC**ATG**TTGCAGCAGGGA**TGA**AAATGAAGAGACAAAGGAAG | ∆*yjjZ* |
| SL4483-yjjZ OL-R | TCATTT**TCA**TCCCTGCTGCAA**CAT**GGTTTCACCTCTGGAATTG | ∆*yjjZ* |
| SL4483-yjjZ-Xma | CCCCCCGGGGTTCCACGACGGAATGCAAAC | ∆*yjjZ* |
| SL1802-1898-Kpn | GGGGTACCGCATTACCGTCTGCGTCC | ∆SL1344_1802 |
| SL1802-1898 OL-F | ACATAA**ATG**AAAAATCCTAAG**TAA**GCAGAATGCTTTTTAAATTC | ∆SL1344_1802 |
| SL1802-1898 OL-R | TTCTGC**TTA**CTTAGGATTTTT**CAT**TTATGTTCTCTGTGTGTTG | ∆SL1344_1802 |
| SL1802-1898-Xma | CCCCCCGGGAAGTCAACGCCCAGACCG | ∆SL1344_1802 |
| sufABCDSE-KOF | GTTCTTAATAAATATCCTGGTGATTTAGAACGCGAGGTAACTCT**ATG**TAGGCTGGAGCTGCTTCG | ∆*sufABCDSE*::kan |
| sufABCDSE-KOR | CGTCCGACAAAGGATTTGACATCAGGTAGAACGCTGTCTGTACAGTCATATGAATATCCTCCTTAGT | ∆*sufABCDSE*::kan |
| fhuB-KOF | CATCCTGAATAACGTGTTGGGAGGCAAAGC**GTG**AGCAGAATGTAGGCTGGAGCTGCTTCG | ∆*fhuB*::kan |
| fhuB-KOR | GGCTGCCACCCGACATAAATGTGGCACAGCTTTTTTCATATGAATATCCTCCTTAG | ∆*fhuB*::kan |
| fepB-KOF | CATTAACTTTATTAATAACAGGACGCTATT**GTG**AGACTCCTGTAGGCTGGAGCTGCTTCG | ∆*fepB*::kan |
| fepB-KOR | AATCGGTCTGGTCAGTCGGATAAGACTCCGATAAGGCATATGAATATCCTCCTTAG | ∆*fepB*::kan |
| nrdHIEF-KOF | ATACGAATC**ATG**AGCATTACTATTTACACTCGCAATAACTGTAGGCTGGAGCTGCTTCG | ∆*nrdHIEF*::kan |
| nrdHIEF-KOR | CCATGCCCGTAAGG**TTA**AAAATTCCAGTCTTCGTCTTCGCATATGAATATCCTCCTTAG | ∆*nrdHIEF*::kan |

Engineered restriction sites are underlined. Start and stop codons are in **bold**.
