# Supplementary material for: Intracellular niche-specific profiling reveals transcriptional adaptations required for the cytosolic lifestyle of Salmonella enterica
Source: PLoS Pathog. 2021 Aug 30;17(8):e1009280. doi: 10.1371/journal.ppat.1009280 (PMC8432900; doi:10.1371/journal.ppat.1009280)
Supplement: S2 Table — (DOCX) [file ppat.1009280.s010.docx]

**S2 Table: Oligonucleotides used for genetic complementation**

| **Name** | **Sequence (5’ to 3”)** | **Plasmid** |
| --- | --- | --- |
| PznuA-XbaF | GCTCTAGAACCAGCAACTGAGGCCTG | pWKS30-*znuA* |
| znuAcomp-rev | CCCAAGCTT**AAT**CTCCTTTCAGGCAGCT | pWKS30-*znuA* |
| soxS-compF | CGGGATCCGGTAATTAGCCCTTTGCTTTC | pWKS30-*soxS* |
| soxS-compR | CGGAATTC**CTA**CAGGCGGTGACGGTAATC | pWKS30-*soxS* |
| Xho-fepBcomp-F | CCGCTCGAGCAAACTGCTGGCGCAATTTC | pGP-Tn7-Cm-*fepB* |
| Xho-fepBcomp-R | CCCCCCGGG**TAA**GGCTAAAACAGGGCGGCA | pGP-Tn7-Cm-*fepB* |

Engineered restriction sites are underlined. Stop codons are in **bold**.
