# Supplementary material for: Intracellular niche-specific profiling reveals transcriptional adaptations required for the cytosolic lifestyle of Salmonella enterica
Source: PLoS Pathog. 2021 Aug 30;17(8):e1009280. doi: 10.1371/journal.ppat.1009280 (PMC8432900; doi:10.1371/journal.ppat.1009280)
Supplement: S3 Table — (DOCX) [file ppat.1009280.s011.docx]

**S3 Table: Oligonucleotides used to construct *gfpmut3* transcriptional reporters**

| Construct | Oligonucleotide sequences (5’ to 3’) | |
| --- | --- | --- |
| P*yjjZ* | PyjjZGFP-XbaF  GC TCT AGA GCC GAC ACG GCG TTG AGA AAC | PyjjZGFP-SmaR  TCC CCC GGG CCG CTG CCT AAC GTC CGC T |
| P*SL1344_1802* | P1802GFP-XbaF  GC TCT AGA TCG CCG TCC ATT TTT ACC TC | P1802GFP-SmaR  TCC CCC GGG AAT AAT GGC TAC GAT AAT AGT TG |
| P*sitA* | PsitAGFP-XbaF  GC TCT AGA CGG CTG ATA GCA GTG CAC | PsitAGFP-SmaR  TCC CCC GGG AGT ATC GCG ACA ATA CCG GC |
| P*sufA* | PsufAGFP-XbaF  GC TCT AGA CTC ATT CAG CAC CTG AAA TGC | PsufAGFP-SmaR  TCC CCC GGG GAA GTC CTC CGG GTT AAA CG |
| P*mntH* | PmntHGFP-XbaF  GC TCT AGA TTT GCT CCA AAT ATG AGG CAG | PmntHGFP-SmaR  TCC CCC GGG CTA TTC TCT ACG CGA TTG |
| P*proV* | PproVGFP-XbaF  GC TCT AGA AAT AGG GAG TCA AAT CGC GC | PproVGFP-SmaR  TCC CCC GGG TCT TTA ACG CCA AGC GAT AGC |
| P*nrdH* | Xba-PnrdH-F  GC TCT AGA TCG GGC GTT CAC GCC GCC | Sma-PnrdH-R  TCC CCC GGG TAA ATA GTA ATG CTC ATG ATT CG |
| P*sopF* | Xba-PSL1177-F  GC TCT AGA CCG CAA TAG TAG TTC ATC C | Sma-SL1177-R  TCC CCC GGG ACC TTT ATA CTT CCA CTA TGG |
| PSTnc3080 | Xba-STnc3080-F  GC TCT AGA CGC CCG CCA TCA CCA CCG GTA | Sma-STnc3080-R  TCC CCC GGG TTT CTC ATT TTA AAT GGA AAT ACG |
| P*iroB* | Xba-PiroB-F  GC TCT AGA GTT GAT ACC ACA GAG ATA GTC | Sma-PiroB-R  TCC CCC GGG ACA AAC AGA ATA CGC ATG AGA |
| P*entC* | Xba-PentC-F  GC TCT AGA TTT GCC GGG GCC AAC CGG | Sma-PentC-R  TCC CCC GGG AGC AAG CGT TGC CAT TGT CT |
| P*sfbA* | Xba-PsfbA-F  GC TCT AGA GGA TGT TGG CGT TAA GAC | Sma-PsfbA-R  TCC CCC GGG AAT GCG TAA ACT CTG ACG CAA |
| P*siiA* | Xba-PsiiA-F  GC TCT AGA GAA TGG TCG GTA TTA TCA | Sma-PsiiA-R  TCC CCC GGG ATT ACT TTC GTC TTC CAT GTT |
| P*ilvC* | PilvC-XbaF  GC TCT AGA CCC GCT TCG GTC AGC GTG | PilvC-SmaR  TCC CCC GGG CAG ATT CAG TGT ATT AAA G |
| P*SL1344_2715* | PSL1344_2715-XbaF  GC TCT AGA GCA TTG CGC GCC AAT GAT | PSL1344_2715-SmaR  TCC CCC GGG CCA GAA GTC ATA ACT ACC |
| P*asnA* | Xba-PasnA-F  GC TCT AGA TGC TGT AGT GAC CGG TGG | Sma-PasnA-R  TCC CCC GGG TTT CAT TTT TTT ACT CCT GCG TC |
| P*cysP* | Xba-PcysP-F  GC TCT AGA GTA TGC GCA GTC CGG TAT TCG | Sma-PcysP-R  TCC CCC GGG TCT CTT TTT CAG TAA GTT AAC GG |
| P*mtr* | Xba-Pmtr-F  GC TCT AGA ACT TCA CGC GTA CCC GCA TTC | Sma-Pmtr-R  TCC CCC GGG AAG CAG CGA AGG TGA CGT TTG GG |
| P*SL1344_3990* | Xba-PSL3990-F  GC TCT AGA TAA TAT GTT CCA CCG ACC CC | Sma-PSL3990-R  TCC CCC GGG AGA CAG GAA AAA TAG TGT TTC ATC |
| P*soxS* | Xba-PsoxS-F  GC TCT AGA TTT CGC AGC GGA CAG TCG C | Sma-PsoxS-R  TCC CCC GGG ATA AGG GTC TGA ATT ATC TGC TG |
| P*uhpT* | uhpT-XbaF  GC TCT AGA ACG GCA ACC GCG GAC CGA TGA | uhpT-SmaR  TCC CCC GGG TCG GCT TGC GCA CCT GGT |
| P*sopE2* | Xba-PsopE2-F  GC TCT AGA CGT CAT GAA AAG TCA TAA TAT | Sma-PsopE2-R  TCC CCC GGG TGG ATA GTG TTA TGT TAG |
| P*sicA* | Xba-PsicA-F  GC TCT AGA AGA CGT GAA GTT CAT ATG | Sma-PsicA-R  TCC CCC GGG ATT ATT TTG ATA ATC CAT TAC |
| P*fhuE* | Xba-PfhuE-F  GC TCT AGA CAT TGA CGG TTG GGA TCA GG | Sma-PfhuE-R  TCC CCC GGG TAT TGA ATG AAA GAC ATC |
| PSTnc3250 | Xba-PSTnc3250-F  GC TCT AGA AGT CAT AGG TAT TGG AAG | Sma-PSTnc3250-R  TCC CCC GGG TAT CAT GCT GCG GTT ATA AAT AAT |
| PSTnc4000 | Xba-PSTnc4000-F  GC TCT AGA GCG TAG ATG GGG CTA CAG | Sma-PSTnc4000-R  TCC CCC GGG TTA ACG CTT CTC CCG TGG CGT |
| P*ygbA* | Xba-PygbA-F  GC TCT AGA TTC AGA CCA TTC GCG AGG ATA | Sma-PygbA-R  TCC CCC GGG ATA CGT TTA CCA GGC ATC GTG |
| P*iroN* | Xba-PiroN-F  GC TCT AGA AAG CGC CTG ATA AAT ATT ACC | Sma-PiroN-R  TCC CCC GGG GAA CTT CTT AAC TCT CAT ACC |
| P*zinT* | PzinT-XbaF  GC TCT AGA AAG CGA GTA GTC ACA AAA ATT ATG CC | PzinT-SmaR  TCC CCC GGG ACT ATT TAC CAA CAG CAT TCC |
| P*mgtC* | PmgtC-XbaF  GC TCT AGA CGT TTA GCA TCC CTT TTC | PmgtC-SmaR  TCC CCC GGG AGC ATA GCG GCC AGT AAA |
| P*fepA* | Xba-PfepA-F  GC TCT AGA AAT AAA ACA GTA GCT GCC GC | Sma-PfepA-R  TCC CCC GGG GAA TGA ATC TTC TTG TTC |
| P*fepB* | Xba-PfepB-F  GC TCT AGA CAA ACT GCT GGC GCA ATT TC | Sma-PfepB-R  TCC CCC GGG AGT CTC ACA ATA GCG TCC TG |
| P*lpxR* | PlpxRGFP-XbaF  GC TCT AGA GGA CAG CCA ACG CTA CAG | PlpxRGFP-SmaR  TCC CCC GGG AAT CAT CGT TGC GCA ATA GC |
| P*trpE* | PtrpE-XbaF  GC TCT AGA CGC TCC GCA ATG AGC CGA | PtrpE-SmaR  TCC CCC GGG TTT TGG TGT TTG CAT GGT |
| P*fhuA* | PfhuAGFP-XbaF  GC TCT AGA TAC GCT GTG CCA GCA GGG C | PfhuAGFP-SmaR  TCC CCC GGG GTG GCT ACT ACA ACT GCG ATT |
| P*grxA* | PgrxA-XbaF  GC TCT AGA CGC CAG CCA GCG GCG TAT TG | PgrxA-XmaR  CCC CCC GGG CAC CCT GGA CGG CCA AAA AT |
| P*fruB* | Xba-PfruB-F  GC TCT AGA CGT CAG GTC AAA CGA TCT C | Sma-PfruB-R  TCC CCC GGG AAC TGG AAC ATG TGT CTC |

Engineered restriction sites are underlined.
