# Supplementary material for: Intracellular niche-specific profiling reveals transcriptional adaptations required for the cytosolic lifestyle of Salmonella enterica
Source: PLoS Pathog. 2021 Aug 30;17(8):e1009280. doi: 10.1371/journal.ppat.1009280 (PMC8432900; doi:10.1371/journal.ppat.1009280)
Supplement: Attachment — Submitted filename: Response to reviewers.docx [file ppat.1009280.s014.docx]

***Part I - Summary*** *Please use this section to discuss strengths/weaknesses of study, novelty/significance, general execution and scholarship.*

***Reviewer #1****: This study reports the niche-specific expression profile of the facultative intracellular pathogen Salmonella Typhimurium. Specifically, upon entry of host epithelial cells, S. Typhimurium is known to inhabit a vacuolar compartment (the SCV), but in some cell types, a small fraction of bacteria escape into the cytosol where they replicate to high numbers; a phenomenon counteracted by host xenophagy (a special form of autophagy). Knodler and colleagues combine experimental induction or inhibition of host autophagy to decrease or increase the cytosolic Salmonella fraction, respectively, with bulk RNA-seq of the recovered intracellular bacteria from the thus treated cell cultures. By comparing genome-wide expression between SCV-enriched and cytosol-enriched bacteria, they define gene sets selectively expressed by intracellular Salmonella dependent on the sub-cellular compartment they occupy. For example, vacuolar Salmonella induce genes that reflect zinc and magnesium limitation. Vice versa, cytosolic pathogens seem to be deprived of iron, upregulated invasion genes required for a second round of infection, and had an expression profile indicative of high proliferation. Using transcriptional reporter fusions in combination with fluorescence microscopy, niche-specific induction of a subset of these genes could be confirmed. What is more, metal ion supplementation assays confirmed the sensitivity of specific gene promoters to the absence of the predicted metal ions. Finally, defined Salmonella deletion mutants were used for infection assays to test for changes in the distribution of vacuolar vs. cytosolic bacteria.
Overall, the definition of the niche-specific transcriptome of this important model pathogen is interesting, relevant and timely. However, I have some technical concerns about the experimental design and analysis, and a couple of comments that I hope will improve this manuscript.*

***Reviewer #2****: In this manuscript, Powers and collaborators successfully established, using RNA-sequencing subsequently validated by transcriptional fusions, the transcriptomic signatures of two Salmonella populations colonizing two different niches within epithelial cells: the cytosol and the vacuole. This impressive resource is very well written, clearly structured, and the hypothesis and results are backed up and put into perspective in a very complete discussion of the literature. To start with, the RNA-seq comparison between cytosolic and vacuolar enriched populations very clearly revealed two distinct virulence genetic programs of the bacteria present in the two different niches highlighted notably by differential metal deprivations (Fe and Mn for the cytosol and Zinc and Mg for the vacuole). The authors then validate the differential RNA-seq signatures using transcriptional fusions of the genes induced in bacteria present in those two niches. Altogether this transcriptional atlas of cytosolic and vacuolar Salmonella reveals what specific program is needed for bacteria to colonize one or the other niche and advances the comprehension of how bacteria can successfully colonize different niches within the host. Therefore, I recommend this paper for publication in Plos Pathogens as the quality of the manuscript and the data presented together with the host-pathogen interaction topic of the manuscript meet the standards of the journal. However, before publication, the authors should answer/take notice of minor concerns listed in the next session.*

***Reviewer #3****:  In this manuscript by Powers et al, the authors start by manipulating the autophagy levels in epithelial cells to shift the intracellular Salmonella population to obtain more (or less, depending on the treatment) cells containing cytosolic bacteria, to further study the transcriptional changes of these bacterial sub-populations by RNA-Seq. The authors then combine their RNA-Seq data with other expression data previously published in the literature, to define genes differentially up regulated either in vacuolar or cytosolic Salmonella. After that, the authors first define and confirm the importance of specific genes related to the differential presence of metals between the cytosol and the vacuole. Then, the authors analyze the expression of cytosol-specific genes that are also up-regulated in late exponential/early stationary phase in vitro. Among these, the authors highlight genes encoding structural genes of the T3SS-1 and some effectors translocated by this system, but also the adhesin SiiE and the lipid-A deacylase LpxR. Having identified the genes that are up-regulated in the cytosol, the authors then go on and use CHQ resistance assays to confirm that the deletion mutants of 5 (of the 18 candidates analyzed) show a reduced proportion of cytosolic bacteria, compared to the wild-type strain. Finally, the authors confirm the results on these 5 candidates using single-cell fluorescence microscopy.

This study is very interesting, as it demonstrates important pathways that define the transcriptional adaptation of Salmonellato these two very different intracellular niches. The experiments are very well-thought and technically sound, and the analyzed phenotypes are very consistent. The main strength is the study of expression of specific genes using single-cell analysis in the context of alterations of the available metals for intracellular bacteria.

The main weakness of the study is the lack of a dynamic study on how the studied pathways affect the intracellular sub-populations, since the authors in general focus on 7-8 hpi, when these sub-populations are already established, but it is not clear if the expression of any of these genes is what drives the establishment of the population or only a consequence. This is particularly interesting in the context of SPI-1 co-regulated genes, since it would be nice to see if the T3SS-1 and effectors are continuously “on” after invasion or if they are truly up-regulated once the bacterium reaches the cytosol.*

Response: We kindly thank the three reviewers for their overall positive assessment of our original submission.

***Part II – Major Issues: Key Experiments Required for Acceptance*** *Please use this section to detail the key new experiments or modifications of existing experiments that should be absolutely required to validate study conclusions.

Generally, there should be no more than 3 such required experiments or major modifications for a "Major Revision" recommendation. If more than 3 experiments are necessary to validate the study conclusions, then you are encouraged to recommend "Reject".*

***Reviewer #1****: • The authors treat the epithelial cell cultures with either EBSS or WTM to induce or inhibit autophagy. Importantly, in both cases treatment of cells extends for 90 min after the bacteria were added to the cultures. Can the authors exclude that EBSS and WTM treatment itself have an effect on Salmonella gene expression? The cleanest way of controlling for this would be to treat an in vitro Salmonella culture with EBSS or WTM, respectively, and sequence their transcriptome. Given that the transcriptomic dataset is such a central part of this study and will likely serve as a resource for the field, I deem this an essential control experiment.*

Response: While we cannot completely exclude that EBSS and WTM will effect *Salmonella* gene expression, we feel it is highly unlikely for the following reasons: (i) EBSS and WTM are removed at 90 min post-infection and samples are collected for transcriptomic analysis at 8 h post-infection, during which time many rounds of bacterial replication will have occurred in both the epithelial cytosol and *Salmonella*-containing vacuole, (ii) the average half-life of a bacterial mRNA is ~5 min (<https://www.pnas.org/content/99/15/9697.long>) and in the 6.5 hours (390 min) after EBSS and WTM washout, the equivalent of 78 mRNA half-lives have passed, (iii) WTM is a phosphatidylinositol 3-kinase (PI3K) inhibitor and *S*. Typhimurium does not encode any known PI3K, (iv) our follow-up analysis using transcriptional reporters validated the niche-specific expression of ~80% of our candidate genes. Importantly this validation was done in untreated epithelial cells i.e. under control conditions in no EBSS or WTM treatments had occurred. Based on these collective arguments, we deemed the suggested “control” experiment would not provide sufficient beneficial information to warrant the costs associated with conducting it.

*• The way the RNA-seq data are analyzed is – at least – uncommon in the field. The authors employ two different tools: RankProduct was originally developed for microarray data and, as far as I know, has previously not been employed to RNA-seq data. BitSeq is a tool to call differentially expressed transcript isoforms in eukaryotes. I do not understand the rationale behind using these two tools, in combination, for the present dataset, rather than using well-established, benchmarked tools that do exist and are commonly used by the community (like edgeR or DESeq). Also, the cutoffs applied seem arbitrary (e.g. fold-change ≤0.71) and are relatively loose (e.g. FDR of 0.2 means that the risk of false discovery is 20%, whereas commonly this cutoff is set to 0.05 for RNA-seq studies). Can the authors please elaborate?*

Response: As suggested, we have now re-analyzed the RNA-seq data for differentially expressed transcripts using DESeq2 v1.30.1. Genes with adjusted p-value of 0.05 or below were deemed differentially expressed. Details are provided in the “RNA-seq data analysis” section of Materials and Methods. The updated RNA-seq data set is included as Dataset S1 and is represented as a volcano plot in Fig 2A of the revised manuscript.

*• In their final experiment, the authors test the ability of defined Salmonella mutants to escape/proliferate in the mammalian cytosol (Fig. 7). In case of four mutants, they claim that the fraction of cytosolic bacteria is significantly lower than in wildtype infections. However, the effects are marginal (whether or not they are indeed statistically significant I cannot assess). Why don’t the authors test the performance of multiple (triple, quadruple) deletion mutants in this assay; this may result in stronger differences (in fact, the authors even mention this possibility in the discussion; lines 472-474). Additionally, judged from Fig. 7B it seems as if the slightly reduced bacterial numbers in the cytosol (in Fig. 7A) are mostly due to a reduced rate of vacuolar escape (rather than lower proliferation in the cytosol). Have the authors tested whether the respective mutants may simply show a delayed vacuolar escape as compared to wildtype bacteria? E.g. how would the result look like when samples are harvested at a later timepoint of infection? Additionally, to test for an intracellular-specific effect, standard growth curves of these mutant strains in rich medium should be reported (as a supplementary figure).*

Response: In Fig 7A of the original submission, mutants that showed a statistically significant decreased proportion of cytosolic bacteria (p<0.05, ANOVA with Dunnett’s post-hoc test) – namely ∆*entC*, ∆*fepB*, ∆*mntH*∆*sitA*, ∆*soxS* – were indicated by red bars and with asterisks. In the revised manuscript, we have expanded the list of bacterial mutants tested and now add ∆*mntH*∆*sitA*∆*entC* and ∆*znuA* bacteria to the list of mutants with statistical significance (new Fig 7A). We would like to point out that an ∆*iroBC* mutant, which is only defective for salmochelin synthesis, does not have a cytosolic proliferation defect, whereas the ∆*entC* mutant cannot synthesize enterobactin and salmochelin, and is defective. Similarly, the ∆*fepB* mutant, which is compromised for cytosolic proliferation, is defective for periplasmic transport of iron delivered via three outer membrane receptors – FepA, CirA and IroN, whereas single *fepA*, *cirA* or *iroN* deletion mutants are comparable to wild type bacteria. Thus, data was presented in our original submission indicating that multiple genetic interruptions in a particular pathway were required to have an overt effect on bacterial proliferation in the cytosol. New data we have included shows that a triple deletion mutant, ∆*mntH*∆*sitA*∆*entC*, which is defective for manganese acquisition and enterobactin and salmochelin synthesis (i.e. two independent pathways), has a greater proliferation defect than the parental ∆*entC* and ∆*mntH*∆*sitA* mutants i.e. an additive effect of genetic deletions (revised Fig 7A). As requested by the reviewer, we have also added data showing that ∆*entC*, ∆*fepB*, ∆*mntH*∆*sitA*, ∆*mntH*∆*sitA*∆*entC*, ∆*soxS* and ∆*znuA* bacteria are not defective for nascent vacuole lysis (revised Fig 7C), nor are they compromised for growth in complex media (Fig S6B). This new data validates that the reduced proportion of bacteria in the cytosol is actually due to the defective proliferation of these mutants this cellular compartment.

***Reviewer #2****: (No Response)*

***Reviewer #3****: Please use this section to detail the key new experiments or modifications of existing experiments that should be absolutely required to validate study conclusions.

Generally, there should be no more than 3 such required experiments or major modifications for a “Major revision” recommendation. If more than 3 experiments are necessary to validate the study conclusions, then you are encouraged to recommend “Reject”

1) The results in this manuscript would benefit from some live microscopy (or at least previous time points) for the expression of genes cytosol- or vacuole-specific. A more dynamic view on the regulation of these pathways would nicely complement the results already shown in this manuscript. This would particularly be interesting for the “SPI-1 associated” genes, as described above. These experiments could easily be done using the gfpmut3constructs.*

Response: We agree with the reviewer about the strengths of live-cell imaging. However, we are unable to conduct such experiments at WSU as our microscopy core facility precludes the use of live infectious bacteria for biosafety reasons. *2) In the last section of the results, the authors use the population-based CHQ resistance assay and mutants in identified genes that are expressed in the cytosol, to evaluate their role in the colonization of this specific niche. The results show that deletion mutants of 5 of the 18 candidates show a reduced proportion of cytosolic bacteria. These results are complemented using the dual-fluorescence reporter pCHAR-Duo(ASV), but only for these 5 candidates. It would be great if the authors can repeat these experiments with one to three of the 13 candidates that did not showed a change in the CHQ resistance assay compared to the wild-type strain, as even the authors comment that the single-cell analysis has a way better resolution than the population-based analysis. On the same line, it would be good to have some complementation assays for at least some the analyzed mutants that show phenotype.*

Response: While we agree with the reviewer that the pCHAR-Duo(ASV) microscopy-based assay has “better resolution” than the CHQ-resistance assay to assess cytosolic and vacuolar replication at the single-cell level, it is also more laborious and subjective than the latter technique. We do blindly count cells containing vacuolar and cytosolic bacteria, and randomly choose fields to count, but scoring 100 infected cells per coverslip is such a small “snapshot” of the entire population that we rarely use this technique for initial screening of phenotypes. Instead we use it as an independent confirmation of other assays. In the revised manuscript, we have added microscopy-based analysis of the *znuA* mutant (Fig 7D, 7E) which surprisingly shows no defect in cytosolic proliferation but enhanced vacuolar replication. We feel that this particular result is a better example of the resolution of this assay rather than including a mutant that has no phenotype, as suggested by the reviewer. Complementation assays have been added to the revised manuscript (Fig 7B). *3) Finally, regarding the pathway enrichment analysis, it would be great if the authors can provide more technical detail on how the analysis was performed, as the authors only link to a webpage containing the scripts. It would be nice to have a more detailed description in the manuscript on how the analysis was made.*

Response: As requested, we have added more details about the pathway enrichment analysis to the Materials and Methods section (lines 800-823).

***Part III – Minor Issues: Editorial and Data Presentation Modifications*** *Please use this section for editorial suggestions as well as relatively minor modifications of existing data that would enhance clarity.*

***Reviewer #1****: • The authors use the term “genes/sRNAs” throughout the manuscript. The reason for doing this remains unclear. The term “gene” includes both coding and noncoding genes. I suggest to replace “genes/sRNAs” with “genes” whenever they speak of expression, and with “mRNAs/sRNAs” whenever they refer to transcripts.*

Response: We thank the reviewer for this clarification. We have made the recommended changes to the terminology throughout the manuscript.

*• At the beginning of the section describing the RNA-seq screen (line 166 ff.), the authors may want to give explicit numbers for the fraction of cytosolic/vacuolar bacteria in the EBSS- or WTM-treated samples that were subjected to RNA-seq. This may be somewhat inferred from Fig. 1C. However, given that the RNA-seq screen is such an integral part of this study, I think this should become clearer as it would otherwise mislead readers to think that the respective samples contained pure populations.*

Response: The proportion of cytosolic bacteria in the total intracellular population in EBSS-treated and WTM-treated conditions has been added to the Results section, lines 162-165.

*• For different assays, the authors use seemingly redundant reporter plasmids for cytosol-exposed bacteria, namely pNF101 and pCHAR-Duo. Could they please explain the rationale of choosing one or the other reporter for the respective experiments?*

Response: pNF101 and pCHAR-Duo(ASV) are both fluorescent reporters of cytosolic access, i.e. bacteria are green when resident in the cytosol. pCHAR-Duo(ASV) does not report on the total bacterial population, however, so pNF101 must be electroporated into bacterial strains harboring chromosomal, constitutively expressed, *mCherry*, in order to identify this particular population. The advantage of the pCHAR-Duo(ASV) plasmid is that it is a dual fluorescent reporter - all bacteria are mCherry-positive and only cytosolic bacteria are GFP-positive. In both cases, *gfp* expression is driven by the *uhpT* promoter and *mCherry* expression by the *trc* promoter. The rationale for when each plasmid was used was dictated by: (i) when the experiments were carried out (pCHAR-Duo(ASV) was constructed a number of years after pNF101 and could be considered a second generation reporter of cytosolic access) and (ii) how many bacterial strains need to be assessed (chromosomal *mCherry* expression requires Tn7-based integration which takes >1 week to complete whereas electroporation takes only 1 day).

*• Likewise, could the authors please elaborate on why they sampled at different timepoints? While the RNA-seq samples (and most other readouts) were taken at 8 h p.i., the CHQ resistance assay samples were instead harvested at 6 h (Fig. 4) and the samples from the infection assay with Salmonella deletion mutants (Fig. 7) at 7 h p.i.*

Response: Samples for RNA-seq and fluorescence microscopy were collected at 8 h p.i., whereas the CHQ resistance assay is performed at 7 h p.i. (not 6 h p.i. as mentioned by the reviewer). We and others have previously shown that the epithelial cells containing hyper-replicating *Salmonella* are undergoing lytic cell death (<https://pubmed.ncbi.nlm.nih.gov/20876119/>, <https://pubmed.ncbi.nlm.nih.gov/25121752/>) and detach very easily from surfaces (<https://pubmed.ncbi.nlm.nih.gov/22719929/>). This this population of bacteria is therefore very easily lost via aspiration i.e. washing of monolayers. The CHQ resistance assay requires a number of media changes/washes and in our hands, more reliable and reproducible results are obtained at 7 h p.i. rather than later timepoints.

*• Strictly speaking, the use of transcriptional reporters (GFP expression from specific promoters) shouldn’t be considered validation of the RNA-seq, since the former measures promoter activity and the latter steady-state transcript levels (i.e. the sum of de novo transcription and RNA decay). Could the authors please rephrase the corresponding sections? Moreover, may this explain why not all of the selected genes could be “validated” by fluorescence reporter assays (Fig. S6)?*

Response: The reviewer brings up an excellent point that we had not considered. We have now removed all references to the fluorescent reporters being used to validate the RNA-seq data in the revised text.

*• Fig. 2: The pathway plot is fine. However, I’d appreciate if the authors also included one panel here that visualizes the entire expression dataset for all Salmonella genes (e.g. a volcano plot [fold-change over significance] or an MA plot (fold-change over abundance]). Given that it is a single comparison (vacuolar vs. cytosolic expression), this shouldn’t take up too much space. I’d further appreciate if mRNAs and sRNAs were colored differently in such a plot and genes that were followed up be labeled by name. Finally, in the pathway plot, the deletion mutants denoted at the left-hand side should be italicized.*

Response: As suggested by the reviewer, we have now included a volcano plot (Fig 2A) to represent the RNA-seq dataset. Genes that we followed up on, as well as other genes that might be of interest to the reader, are indicated. The deletion mutants listed on the left-hand side of the pathway plot (Fig 2B) are now italicized.

*• Fig. 7A: The plot contains data for a sitA/mntH double knockout (which is associated with a slightly reduced fraction of cytosolic bacteria). The two single mutants (delta-sitA and delta-mntH) should be included to this assay to test which of the two genes is responsible for this (subtle) effect.*

Response: Data for the ∆*mntH* and ∆*sitA* mutants is included in Fig 7A of the revised manuscript. Neither mutant is defective for the proportion of cytosolic bacteria.

*• I recommend the authors to include a model figure that summarizes the major findings revealed in this work, comparing Salmonella in the SCV and in the cytosol.*

Response: A model figure has been included as Figure 8 in the revised manuscript.

*• Methods: A section is missing that describes how the cDNA libraries were prepared and how the sequencing was performed (read length, single- or paired-end mode, etc.).*

Response: This information has been added to the “Bacterial RNA enrichment from infected cells, cDNA synthesis and Illumina sequencing” section of the Materials and Methods section of the revised manuscript (lines 763-776).

*• References: Some of the references seem to be skewed (examples include Ref #116, #122, #128).*

Response: We are unclear as to what the reviewer exactly means by this comment. Below we explain why we cited these particular references in the original and revised manuscript but also acknowledge that our explanation might not address the particular concern that the reviewer has. Sturm *et al*. (2011) (<https://pubmed.ncbi.nlm.nih.gov/21829349/>) (ref 116 in original manuscript, ref 80 in revised manuscript) was referenced to support of our findings about the variability in expression levels of SPI1-associated genes for cytosolic bacteria. We have cited the results of Liu *et al*. (2015) (<https://pubmed.ncbi.nlm.nih.gov/25939512/>) (ref 122 of original manuscript, ref 136 of revised manuscript) to draw attention to conclusions that were made about metal ion acquisition by intracellular *S*. Typhimurium without taking into account the bacterial sub-populations in epithelial cells. The single gene deletion library of *S*. Typhimurium mutants created by Michael McClelland and Helene Andrews-Polymenis (<https://pubmed.ncbi.nlm.nih.gov/19578432/>, <https://pubmed.ncbi.nlm.nih.gov/25007190/>) (uncited and ref 128 in original manuscript, references 142 and 143 in revised manuscript) was the source of many of the bacterial gene deletion mutants for this study.

*Text edits:
• Lines 228-231: The sentence seems grammatically incorrect.*

Response: Sentence has been corrected (lines 233-234 of revised manuscript)

*• Line 301: “Compared to untreated cells at Fig 6 h p.i. (…)” Please remove “Fig”.*

Response: Sentence has been corrected (lines 311-312).

*• Line 346: A verb is missing from this sentence.*

Response: Sentence has been updated to “*S*. Typhimurium also has multiple transporters for Mg^2+^ uptake” (line 360 of revised manuscript).

*• Line 478: “(…) mutants fail to proliferate in the epithelial cytosol” This seems to be an overstatement. As described in my above comment, from the data in Fig. 7 I rather conclude that the mutants are (slightly) less efficient in escaping from the vacuole and entering the cytosol. In contrast, to me these data do not imply that the mutants fail to proliferate.*

Response: We have new data indicating that all of the identified mutants act like wild-type bacteria for lysis of their nascent vacuole (Fig 7C of revised manuscript), therefore they are not “less efficient in escaping from the vacuole” as suggested by the reviewer. Regardless, we do agree with the reviewer that we had originally overstated the phenotype of these mutants and have revised the text to state “….mutants have impaired cytosolic replication abilities” (line 519 of revised manuscript).

*• Line 501: “S. flexneri does not encode for mgtB.” This statement sounds redundant as already the previous sentence says that.*

Response: The redundant sentence has been removed from the revised text.

*• Line 578: “(…) deleted for amino acids 4-Fig 66) and (…)” Please correct.*

Response: Sentence has been corrected (line 633 of revised manuscript).

*• Line 596: “(…) replacement was by PCR (…)” It seems like a verb is missing.*

Response: Sentence has been corrected to “The correct integration of the kanamycin resistance cassette was confirmed by colony PCR with a target-flanked primer and a kanamycin resistance gene-specific primer.” (lines 650-652 of revised manuscript.

*• Line 749: “(…) confocal microscope was using the (…)”It seems like a verb is missing.*

Response: Sentence has been corrected to “Images were acquired using confocal microscopy (Leica SP8) in sequential acquisition mode through an optical section of 0.3 µm in the z-axis”. (lines 849-851 of revised manuscript).

***Reviewer #2****: 1. The choice of EBSS to deplete cytosolic bacteria (and therefore to enrich the vacuolar population) and WTM to enrich cytosolic bacteria is disputable:
Can the authors comment why they did not sort the cytosolic bacteria using for example their pNF101 plasmid and perform RNA-seq on a sorted population which would have been neater as opposed to result in a mix between cytosolic and vacuolar bacteria (see fig 1D; after 8h pi in presence of WTM, the vacuolar bacteria are still present)? I agree that this fraction seems to be the same in the EBSS treatment and that the comparison between EBSS and WTM treatments provides the authors with their “up cytosol” genes but an RNA-seq on sorted populations would have been better.*

Response: We initially used fluorescence-assisted cell sorting experiments in our attempt to separate uninfected epithelial cells from infected epithelial cells containing either vacuolar or cytosolic *Salmonella*. The roadblock that we quickly hit is that, while cells with vacuolar bacteria are easy to sort in a short period of time, cells filled with cytosolic *Salmonella* are not. They are a minor proportion of the total epithelial population (~10% of infected cells and <50% of the epithelial population is infected) and very fragile. Increasing the flow rate to shorten the sorting time caused the pyroptotic epithelial cells to burst whereas reducing the flow rate to keep these cells intact took too long (>1 h) to sort sufficient infected cells into Trizol for downstream analysis, knowing that the half-life of bacterial mRNAs is ~5 min. As a result, we deemed this approach unsuitable after ~ 6 months of our efforts.

*2. Line 145 / Line 409. The authors are using two different Gfp (Ova and ASV), one being more stable than the other. Please explain.*

Response: pNF101 was constructed in the lab of Dirk Bumann and utilizes *gfp(ova)* as a fluorescent reporter of the intracellular location of *S*. Typhimurium. GFP(ova) has a protein half-life of ~60 min. pCHAR-Duo(ASV) was constructed in our lab and utilizes *gfp(ASV)* as a reporter of cytosolic access of *Salmonella*. This GFP variant has a slightly longer half-life of ~110 min. Both are regarded as “high-turnover” GFP variants, compared to GFPmut3 for example (half-life of ~24 h). The parental plasmid of pCHAR-Duo(ASV) is pCHAR-Duo and was constructed in the Steele-Mortimer lab (<https://onlinelibrary.wiley.com/doi/10.1111/mmi.14361>). In our hands, all bacteria harboring this particular plasmid are GFP-positive, regardless of whether they were in the cytosol or SCV. This plasmid has the advantage over pNF101 of producing mCherry fluorescence in all bacteria. Given that cytosolic *S*. Typhimurium can be captured by autophagy or repair their damaged vacuole, we felt that destabilized GFP would be a more accurate reporter of bacteria that were accessible to the cytosol, or had recently been, rather than GFPmut3, which can only report on bacteria that either were, or had at one time, accessed the epithelial cytosol. We have extensive experience using the (LVA), (AAV) and (ASV) destabilized variants of GFP (<https://pubmed.ncbi.nlm.nih.gov/20035008/>, <https://pubmed.ncbi.nlm.nih.gov/29208746/>) in *S*. Typhimurium and chose to replace *gfpmut3* in pCHAR-Duo with *gfpmut3(ASV)* to create a more accurate sensor of cytosolic access. We have no indication that quantification of cytosolic *S*. Typhimurium differs when comparing results with pNF101 or pCHAR-Duo(ASV) and routinely use both of these reporters in our lab.

*3. Line 167 – 169. The authors refer to the WTM treated bacteria as “enriched for vacuolar Salmonella” but contrarily to EBSS where a clear enrichment of the cytosolic fraction is seen, it is not the case for the vacuolar fraction in the WTM condition. There, what is seen is more a depletion of the cytosolic bacteria than an enrichment of the vacuolar bacteria. The authors should therefore rewrite this specific sentence accordingly. In addition, I believe that instead of “enriched for either cytosolic or vacuolar S. Typhimurium by EBSS-or WTM treatment”, we should read “enriched for either cytosolic or vacuolar S. Typhimurium by WTM – or EBSS treatment”.*

Response: We agree with the reviewer that our descriptors for these two populations was not entirely accurate. In the revised manuscript, we now state “To define the transcriptional signature of cytosolic *S*. Typhimurium, we isolated bacterial RNA from epithelial cells that had been treated with EBSS or WTM, conditions that depleted or enriched the cytosolic population, respectively” (lines 172-174). We trust the reviewer will agree that this is a more accurate description.

*4. It is unclear to me why the authors chose to compare directly WTM and EBSS conditions rather than compare WTM with untreated; EBSS with untreated and the results of those two comparisons compared to each other. Please explain.*

Response: The decision to not include control conditions in the RNA-seq analysis was based on two factors: (i) we did not feel adding two more biological replicates from untreated samples would produce sufficient informative data to justify the additional costs, (ii) we wanted to compare conditions that gave the largest difference in the proportion of cytosolic bacteria to maximize our chances of identifying differentially expressed genes. In the end, we are glad that we chose to not prepare untreated samples for transcriptomic analysis as the range of fold-change in gene expression comparing EBSS and WTM treated samples (the two extreme conditions) was only 0.17-3.25-fold.

*5. I do miss p-values on the panels for each figure, especially in figure 4B but also throughout the manuscript. Differences are sometimes difficult to appreciate and the number of repeats is very low (n=2). Another example of small but significant differences where p-values on the graph are needed, is figure 7 panel A (even though here, the number of repeats (more than 5) makes the data stronger.*

Response: Statistically significant data points are now shown in revised Figures 1, 4, 7. We used two statistical tests to compare data: (i) ANOVA with Dunnett’s post-hoc analysis for data with a normal distribution and (ii) Kruskal-Wallis test for data with a non-normal distribution. In both cases p<0.05 was considered significant. Details are provided in the “Statistical analysis” section of Materials and Methods (lines 854-859 of revised manuscript). Even though some experiments were repeated only twice, we have changed the graph style to incorporate the mean from each experiment and thereby illustrate how little variability there is between independent experiments. See Fig 3 of the revised manuscript for example.

*6. T0 controls (before infection or even in the ON culture) are missing for the following panels: fig3, lower panel; fig 4, panels A and B; fig 5 lower panel; fig 6 lower panel. If the authors performed those experiments, it would be a plus to display them.*

Response: We thank the reviewer for this excellent suggestion and have now completed Image J analysis of GFP signal intensity for bacteria grown to late-log phase in liquid broth (t_0_ samples). See Fig 3, Fig 5, Fig 6 and Fig S7 of the revised manuscript.

*7. Line 275, fig 4B. Did the authors perform any growth curves experiments to access the toxicity of DPI (especially since DPI affects replication, line 298). If DPI itself is toxic for bacteria, it could explain the Gfp accumulation observed in fig 4B. Please comment.*

Response: We have now added growth curve data for bacteria in complex media (LB-Miller broth) in the presence of 200 µM DPI or vehicle (ethanol) control (Fig S6A). DPI has a negative effect on bacterial growth, reducing the doubling-time during log phase and the culture density in lag phase. Other studies have shown that this growth inhibition is reversible upon the addition of an iron source (<https://journals.asm.org/doi/abs/10.1128/iai.57.4.1271-1275.1989>, <https://journals.asm.org/doi/10.1128/iai.64.11.4549-4556.1996>) indicating that this effect is not due the toxicity of DPI itself, but rather its chelation of iron (and likely other metals given its broad specificity).

*8. Line 290, fig 4A. A non-iron related gene as a control would have been a great addition. Again, if the authors have done such control, it would reinforce the trends observed in panel A after the gradual addition of FAC.*

Response: These FAC titration experiments were designed to confirm the *in vivo* responsiveness of transcriptional reporters to metals in an infection context. Due to the large number of samples, combined with the time that the imaging and analysis entails, we only included those reporters whose expression was affected by iron in minimal media conditions. The means from each experiment are now shown in the revised Figure 4A and the consistency in results from individual experiments is clear, reinforcing sample-to-sample reproducibility in the observed trends.

*9. Typo mistakes (twice, line 301 and 302 for 8 hpi).*

Response: The typo has been corrected (line 312 in revised manuscript)

*10. Line 346. Is a verb missing?*

Response: Sentence now reads “*S*. Typhimurium also has multiple transporters for Mg^2+^ uptake” (line 359 of revised manuscript)

*11. Overall, the hypotheses are all very well supported by the data, with the exception of Mn. I would value the results of a transcriptional fusion only dependent on Mn. I believe the two the authors mentioned have been tested for iron experiments earlier in the manuscript and since those genes are dependent on both iron and Mn, the investigations of the authors stopped there for Mn. Did the authors find an “up cytosolic” gene only dependent on Mn that they could have tested instead?*

Response: MntR serves as the primary sensor and transducer of manganese abundance in enterobacteria. In *S*. Typhimurium, *mntH* and *sitABCD* are the only genes known to be regulated by MntR. *mntH* and *sitABCD* are also transcriptionally regulated by Fur (<https://pubmed.ncbi.nlm.nih.gov/15659669/>, <https://pubmed.ncbi.nlm.nih.gov/12029030/>, <https://pubmed.ncbi.nlm.nih.gov/11466284/>), which explains their sensitivity to both manganese and iron (Fig S5). In lab strains of *E. coli* (which do not encode *sitABCD*), the MntR mini-regulon is comprised of only five genes: *dps*, *mntH*, *mntP* (*yebN*), *mntR* and *mntS* (*rybA*) (<https://pubmed.ncbi.nlm.nih.gov/21908668/>, <https://pubmed.ncbi.nlm.nih.gov/21239586/>) - *dps*, *mntP*, *mntR* (SL1344_0810) and *mntS* are constitutively expressed in vacuolar and cytosolic *Salmonella* according to our RNA-seq data (Dataset S1). Therefore, *mntH* and *sitABCD* appear to be the only manganese-sensitive genes in *S*. Typhimurium that are up-regulated in the cytosolic population. The caveat is that their transcription is also regulated by iron concentration, as the reviewer correctly states. Due to overlap in the binding sites of Fur and MntH, it is not possible to genetically engineer the promoter regions of *sitA* or *mntH* to prevent the binding of one of these transcription factors (<https://pubmed.ncbi.nlm.nih.gov/15659669/>).

*12. Line 359. Please add a conclusion to this paragraph regarding all the findings on metal in the two niches.*

Response: As suggested, a conclusion has now been added for this paragraph (lines 372-375 of revised manuscript).

*13. Line 428. Please add a reference for the pCHAR-Duo (ASV).*

Response: The dual reporter plasmid, pCHAR-Duo(ASV), was constructed in our lab for this study. A description of its construction is provided in the Materials and Methods (lines 676-682).

***Reviewer #3****: Please use this section for editorial suggestions as well as relative minor modifications of existing data that would enhance clarity.

Lines 123-124: “here we define the niche-specific…” the authors only define some of the environments encountered by S. Typhimurium in these niches.*

Response: Sentence has been modified to the following “….here we describe niche-specific environments encountered by *S*. Typhimurium in epithelial cells….” to more accurately reflect that we have not completely defined these two environments (lines 124-126 of revised manuscript). *Line 130: the authors mention genetic manipulation of autophagy levels; can they show any data showing that a non-nutritional upregulation of autophagy reduces the fraction of cytosolic bacteria?*

Response: We chose nutrient starvation (EBSS) as it is a rapid and accepted means of activating the autophagy pathway that occurs via well-defined signaling pathways. Chemicals and small molecules can also activate autophagy, albeit via less well-defined mechanisms. Chu *et al*. (2020) have shown that butyrate-mediated autophagy induction limits the cytosolic replication of *S*. Infantis in colonic epithelial cells (Caco-2) (<https://veterinaryresearch.biomedcentral.com/articles/10.1186/s13567-020-00823-8>), results similar to our own in HeLa epithelial cells. Another study identified that trifluoperazine (TFP), a phenothiazine derivative that activates autophagy, suppresses *S*. Typhimurium growth in HeLa cells, although whether this effect was on cytosolic and/or vacuolar bacteria was not investigated (<https://pubmed.ncbi.nlm.nih.gov/23973919/>). Together, these independent studies illustrate that activation of autophagy has limiting effect on *Salmonella* replication in epithelial cells, irrespective of the stimulus.

*Line 139: first appearance of EBSS, please define it here. It is only defined in line 663.*

Response: EBSS is now defined upon its first appearance in the text (line 141 of revised manuscript). *Lines 175-176: how were the Fold-change values defined?*

Response: The mean TPM for EBSS and WTM treatment was averaged from two independent, i.e. biological, replicates. Fold-change was then calculated as mean TPM for WTM treatment/mean TPM for EBSS treatment (see Dataset S1). *Lines 228-230: the authors highlight the use of GFP as an “ideal reporter to study differential gene expression”, but they don’t explain why they specifically use the gfpmut3 variant*

Response: We chose to use *gfpmut3*-based reporters in *S*. Typhimurium based on two criteria: (i) it has successfully been used to study intracellular gene expression previously, (ii) many of the transcripts of interest were expressed at low levels (<100 TPM, Dataset S1). Using a single fluorescent reporter, we wished to test as many genes of interest as possible and maximize the likelihood of generating *Salmonella* that were sufficiently fluorescent to detect by microscopy. Based on our past experience, this requires either high copy number expression (which is detrimental to the intracellular fitness of *Salmonella*) of destabilized GFP, or low copy number expression of stable GFP or chromosomal integration (laborious to construct for the number of genes we tested). We have explained in more detail in the revised manuscript our rationale for choosing *gfpmut3* (lines 236-239). *Line 380: LpxR is not a hypothetical protein, but a 3’-O-deacylase of the Salmonella lipid A (see Reynolds et al 2006 DOI: 10.1074/jbc.M603527200 and Kawasaki et al 2012 doi: 10.1016/j.bbrc.2012.10.054). Even more, is quite interesting that an enzyme that modifies the lipid A reducing its ability to activate the immune system, is up-regulated in the host cytosol, but the study of this is beyond the scope of this manuscript.*

Response: The suggested publications have now been cited (line 400 of revised manuscript) *Line 578: looks like there is a typo in the yjjZ deleted amino acids “4-Fig 66”*

Response: The typo has been corrected (line 633 of the revised manuscript). *Figure 3, 5 and 6: to make it more consistent, maybe consider labelling the single channels with the fluorescence shown instead of having a mixture (GFP and STm)*

Response: As suggested, “GFP” and “STm” have been replaced with “GFP” and “mCherry” in all relevant figures (Fig 3, Fig 5, Fig 6, Fig S3, Fig S4, Fig S7, Fig S8 of the revised manuscript).
